# A 16q deletion involving *FOXF1* enhancer is associated to pulmonary capillary hemangiomatosis

**DOI:** 10.1186/s12881-015-0241-7

**Published:** 2015-10-13

**Authors:** Patrizia Dello Russo, Alessandra Franzoni, Federica Baldan, Cinzia Puppin, Giovanna De Maglio, Carla Pittini, Luigi Cattarossi, Stefano Pizzolitto, Giuseppe Damante

**Affiliations:** Dipartimento di Medicina di Laboratorio, Azienda Ospedaliero-Universitaria S. Maria della Misericordia, Udine, Italy; Dipartimento di Scienze Mediche e Biologiche, Università di Udine, Piazzale Kolbe 4, 33100 Udine, Italy; Dipartimento Materno-infantile, Azienda Ospedaliero-Universitaria S. Maria della Misericordia, Udine, Italy

**Keywords:** Pulmonary capillary hemangiomatosis, Chromosomal abnormalities, Deletion, Gene regulation

## Abstract

**Background:**

Pulmonary capillary hemangiomatosis (PCH) is an uncommon pulmonary disorder, with variable clinical features depending on which lung structure is affected, and it is usually linked to pulmonary arterial hypertension. Congenital PCH has been very rarely described and, so far, the only causative gene identified is *EIF2AK4,* which encodes for a translation initiation factor. However, not all PCH cases might carry a mutation in this gene.

**Case presentation:**

We report the clinical and cytogenetic characterization of a patient (male, newborn, first child of healthy non-consanguineous parents) died after three days of life with severe neonatal pulmonary hypertension, due to diffuse capillary hemangiomatosis diagnosed post mortem.

Conventional karyotyping, Microarray-Based Comparative Genomic Hydridization (CGHa) and quantitative PCR were performed. CGHa revealed a heterozygous chromosome 16q23.3q24.1 interstitial deletion, spanning about 2.6 Mb and involving a *FOXF1* gene enhancer. Quantitative PCR showed that the proband’s deletion was *de novo*. Microsatellite analysis demonstrate that the deletion occurred in the maternal chromosome 16.

**Conclusion:**

*FOXF1* loss of function mutation have been so far identified in alveolar capillary dysplasia with misalignment of pulmonary veins (ACD/MPV), a lung disease different from PCH. Our data suggest the hypothesis that disruption of the *FOXF1* gene enhancer could be a genetic determinant of PCH. Moreover, our findings support the idea that *FOXF1* is a paternally imprinted gene.

**Electronic supplementary material:**

The online version of this article (doi:10.1186/s12881-015-0241-7) contains supplementary material, which is available to authorized users.

## Background

Pulmonary capillary hemangiomatosis (PCH) is a rare disorder that was first reported in 1978, with less than a few hundred nonrelated cases reported, so far [1]. The PCH frequency within the general population is actually unknown [2, 3]. PCH anatomopathological features are pulmonary hypertension and excessive neovascularization characterized by capillary-sized blood vessels within the pulmonary interstitial tissue, vasculature, and airways [4, 5]. Clinically, PCH cases are quite variable; also because, this disease could mimic different lung diseases, including pulmonary veno-occlusive disease, idiopathic pulmonary arterial hypertension and atypical interstitial lung disease [2]. Nowadays, the only genetic cause identified is a mutation in *EIF2AK4* gene *(Eukaryotic translation initiation factor 2 alpha kinase 4, in chromosome 15q15.1)*, which encodes for a translation factor [6]. However, not all PCH cases might carry a mutation in this gene [2].

We report the clinical and genetic characterization of a newborn male presenting early severe pulmonary hypertension, due to a post-mortem diagnosis of PCH.

He carried a 2.6 Mb sized 16q23.3q24.1 deletion, as demonstrated by CGHa. He displayed no additional anomalies or malformations, and no familial occurrence.

## Case presentation

### Clinical report

The patient was the first child of nonrelated healthy parents with a negligible familial history. The patient was a full term (41 weeks) male infant, born by induced vaginal delivery to a 38-year old mother, whose pregnancy was uneventful. Birth weight, length and head circumference were 3340 gr, 50.2 cm and 34.0 cm, respectively. Apgar scores were 4-10-10. After birth, since the patient did not show spontaneous breathing and was hypotonic, he was stimulated and ventilated by ambu (FiO_2_: 0.4). Because he presented an increased O_2_-requirement after one hour of life, he was subjected to oxygenation by FiO_2_ 40 %. Chest radiography showed widespread hypodiafania of the right hemithorax. Blood tests were negative for infection. Ultrasound revealed a normal brain anatomy, with mild hyperechoic periventricular left rear. Ultrasonography was compatible with wet lung disease. 21 h after birth, the infant developed a deteriorating respiratory distress and echocardiography documented a severe pulmonary hypertension in the absence of congenital structural abnormalities. He died at the third day of life from respiratory distress, pulmonary hypertension, heart failure and an extreme bradycardia. The autopsy demonstrated a lung parenchyma characterized by marked edema and stasis.

### Materials and methods

#### Histology and immunohistochemistry

Tissue from autoptic lung samples were fixed in 4 % formalin for about 24 h. Fixed tissue was then paraffin embedded and 4 μm slides were stained with hematoxilin and eosin. Immunohistochemical stains for CD31 (1:200, clone JC70A, Dako, Denmark), CD34 (1:50, clone QBEND10, Dako, Denmark) and histochemical Masson’s trichrome stain were performed for vascular pattern observation.

#### Cytogenetics and molecular genetics

Blood samples of proband and parents were obtained after a signed informed consent. Parents informed consent was also given to analyse their own genomes. We explained that analysis of the parents would be useful to better understand the proband results. When CGHa is performed, obtaining blood samples from parents is part of our standard care.

Conventional high resolution karyotyping (GTG banding) was performed on proband blood lymphocytes following conventional procedures.

For CGHa analysis genomic DNA was isolated from an EDTA peripheral blood sample using QIAamp Blood Midi Kit according to the manufacturer procedure (Qiagen, Hilden, Germany). Molecular karyotyping was performed in accordance with the manufacturer procedure with an 180,000-oligonucleotide microarray (Sure Print G3 Human CGH Microarray Kit 180 k, Agilent Technologies, Santa Clara, CA, USA). The genomic sample was labelled and hybridized according with the Agilent Enzymatic Labelling protocol. We used a male DNA Coriell GM10851 (Coriell Institute, Camden, NJ, USA) as a normal reference. CGH Agilent Genomic Workbench Lite Edition 6.5.0.18 software and UCSC hg19 assembly were used to analyze the results. The presence of a copy number variation was defined by the presence of an abnormal log2 ratio for at least three contiguous oligonucleotides. The presence of the deletion in our patient was confirmed by quantitative PCR using 7300 Real Time PCR System (Applied BioSystems, Foster City, CA, USA) (data not shown). Primers inside *OSGIN1, EMC8, ZDHHC7* genes were employed.

Microsatellites located into the deletion were analyzed by PCR and capillary electrophoresis sizing of the products. Microsatellites were amplified using the following primers:

- *L17941*, Forward: 5′- 6FAM-CTGGGTACTCTTCTGTGACA-3′;
- Reverse: 5′-CTCTCTCCCCAACATGGTG-3′.
- *L29692*, Forward: 5′-6FAM-TGTGTGTCTTCTGGGGGAGT-3′;
- Reverse: 5′-CACAGCTAGCCACAGGCAG-3′

The amplified products were analyzed by capillary electrophoresis (3500 IDX sequencer, Life Technologies).

### Results

**Fig. 1:**
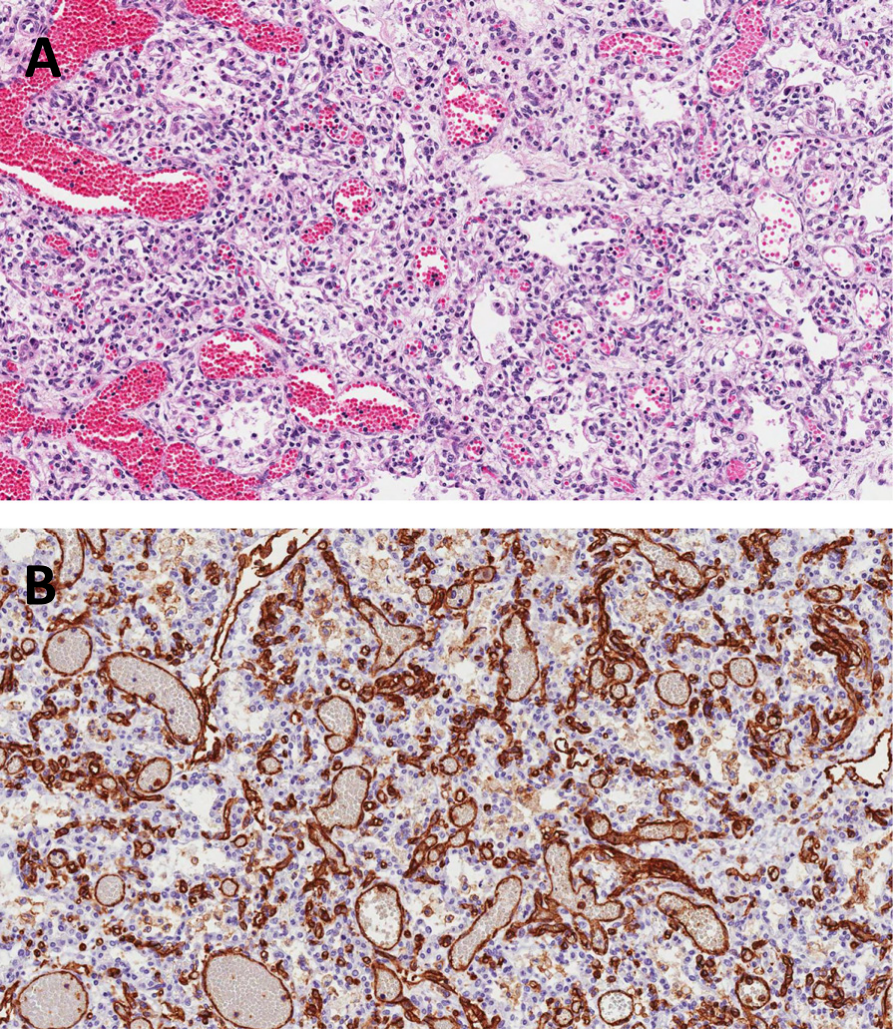
Microscopic evaluation of lung tissues. Panel **a**, histologic image (Hematoxilin-Eosin, 20X) of lung autoptic sample showing widespread proliferation of septal capillaries with extensive alveolar hemorrhage, without a proliferation in septal, or lung bronchovascular bundles. Panel **b**, CD31 endothelial immunostaining (20X) demonstrates marked septal capillaries dilatation and proliferation

Histopathological analysis of the lungs showed marks of PCH (Fig. 1, panel a). Sections of the lung reveal lobular architecture with acinar underdevelopment and numerous dilated vascular channels within the thickened septa. Reduced radial-alveolar count was detected, reflective of decreased alveolarization. Air-blood barriers were detected within the alveolar walls; however, a rich, congested, proliferated capillary network was present within interalveolar septa. This is associated with some hemorrhage into the airspace as well as some regions with large number of hystiocytes in the airspaces. Preacinar and intra-acinar arteries had thick smooth muscle medial coats and there was an extension of smooth muscle into the peripheral intra-acinar arteries up to the alveolar wall level. In addition, a thick collagen adventitial coat was present around the preacinar arteries. These structural features, are reminiscent of those present in persistent pulmonary hypertension of the newborn. Abnormalities of pulmonary lymphatics were not present. No pulmonary venous misaligment or obstructive/occlusive changes are discerned. Immunohistochemical analysis of CD31 demonstrates septal capillaries dilatation and proliferation (Fig. 1, panel b). Altogether, these features indicate the presence of an intractable hypertensive pulmonary vascular disease of the newborn consistent with the morphologic characteristics of pulmonary hemangiomatosis with structural abnormalities in preacinar and intra-acinar pulmonary arteries (“post-resistant segment of the vasculature”).

Standard karyotyping showed no abnormalities (46,XY).

**Fig. 2:**
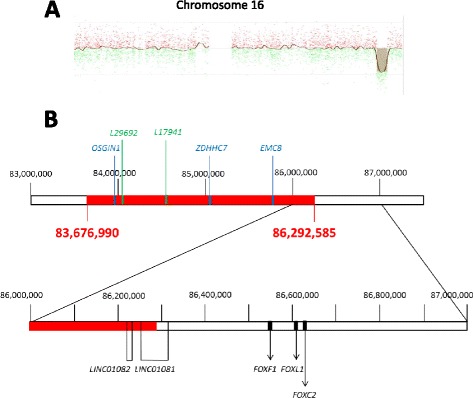
CGHa analysis. Panel **a**, CGH signals of patient’s chromosome 16. The deleted region is highlighted by a brown area. Panel **b**, chromosome 16 region containing the deletion. The deleted region is shown in red. Positions of FOX genes are shown in black. Positions of microsatellites *LINC01081* and *LINC01082* utilized for analysis are shown in green. Location of genes utilized for quantitative PCR are shown in blue. Base-pairs are numbered according to hg19

CGHa identified a submicroscopic deletion in 16q23.3q24.1 cytoband (deletion size 2.6 Mb) (Fig. 2). The deletion spans from 83,676,990 to 86,292,585 bp, encompassing LINC01082 and disrupting LINC01081, which are long non-coding RNAs located in the FOXF1 tissue-specific distant enhancer, mapping 0.3 Mb upstream FOX genes cluster [7]. Positions are referred to Genome Assembly hg19. In the Additional file 1: Table S1 all genes contained in the deletion are listed. Among them, only the *DNAAF1* gene deficiency is known to cause a lung disease, i.e. the primary ciliary dyskinesia (or Karatgener syndrome), in which, however, airways cilia, but not alveoli and their vessels, are affected.

**Fig. 3:**
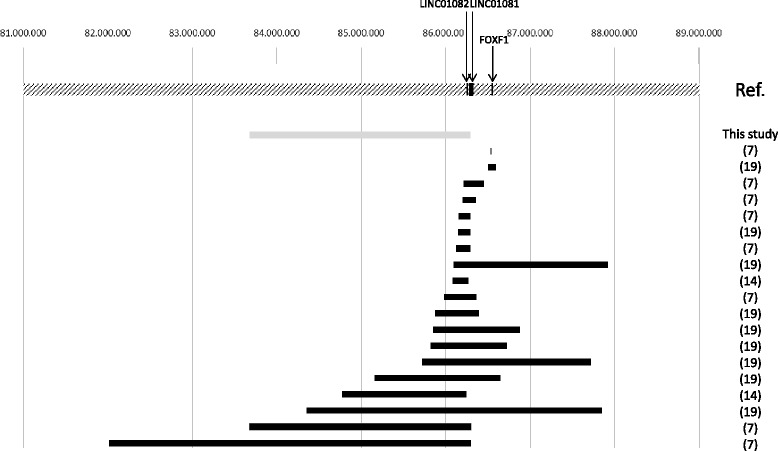
Deletions associated with PCH and ACD/MPV. The map of the FOXF1 region is represented at the top: the FOXF1 gene and the long non-coding RNAs LINC01081 and LINC01082 are shown. The gray bar below indicates the deletion found in our PCH patients, while black bars indicate deletions in patients with ACD/MPV so far found

No other pathogenetic genomic imbalance was detected in the proband sample. Deletions in this region have been recently found in patients with Alveolar Capillary Dysplasia with Misalignment of Pulmonary Veins (ACD/MPV) [7]. In Fig. 3 are shown all deletions of the *FOXF1* region so far identified in patients with ACD/MPV. PCH and ACD/MPV are two different diseases related to opposite phenomena in lung development disruption. In fact, PCH is characterized by capillaries proliferation in pulmonary interstitium [4, 5], while in ACD/MPC immature lobular development and reduced capillary density are present [8, 9]. In our patient, acinar underdevelopment and decreased alveolarization were present, which are reminiscent of ACD/MPV. However, we did not find the other typical feature of ACD/MPV, i.e. misalignment of pulmonary veins, in our case. Instead, the major histological finding of our patient was the capillary proliferation within the interalveolar septa, which is typical in PCH.

By quantitative PCR, our patient parents were evaluated: both subjects presented no abnormalities, thus suggesting the *de novo* origin of the deletion.

**Fig. 4:**
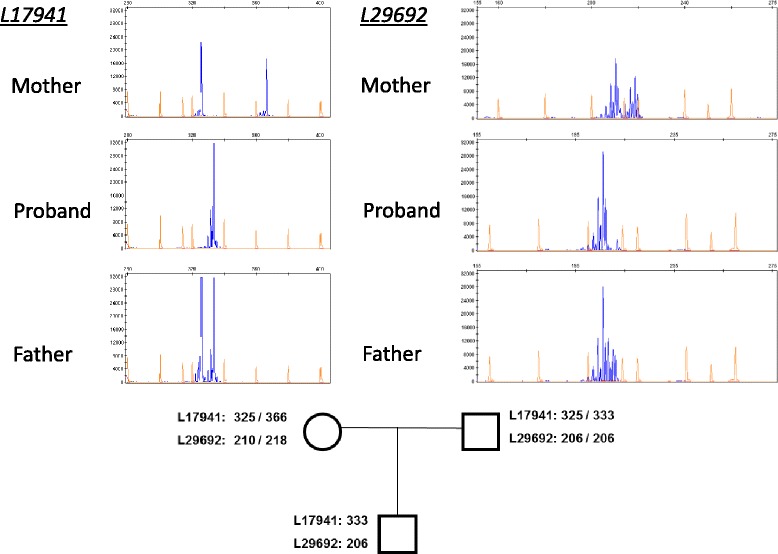
Microsatellite analysis. *Top*, electropherograms of *L17941* (*left*) and *L29692* (*right*). *Bottom*, pedigree of the family with alleles of *L17941* and *L29692* present in each subject. Numbers correspond to the base-pair length of each allele

It is known that the 16q23.3q24.1 region could be subjected to parental imprinting; in fact, in ACD/MPV, the deletion always occurs in the maternal chromosome and the paternal allele is less expressed than the maternal one [10]. Thus, in order to test whether the 16q23.3q24.1 deletion occurred in the maternal chromosome, a microsatellite analysis was conducted inside the deletion. As shown in Fig. 4, analysis of L17941 and L29692 microsatellites indicate that the deletion indeed occurs in the maternal chromosome.

## Conclusion

The PCH histopathologic lungs features show a crowded and congested alveolar capillary bed without pulmonary venous misalignment and lymphatic alteration. The alveolar capillary expansion is usually associated with intraalveolar hemorrhage [4, 5]. Based upon our microscopical findings, our patient suffers of PCH with structural abnormalities in preacinar and intra-acinar pulmonary arteries, consistent with the morphologic characteristics of persistent pulmonary hypertension of the newborn. The radial-alveolar counts were reduced, reflective of decreased alveolarization.

PCH is considered an underestimated pathology, because it may mimic idiopathic pulmonary arterial hypertension, pulmonary veno-occlusive disease, atypical interstitial lung disease, misaligment of lung vessel and alveolar capillary dysplasia or congenital pulmonary lymphangectasia [2, 11–13]. Based on familial occurrence, it is possible that congenital forms of this disease may have a genetic determination. Best et al. [6] have shown the involvement of *EIF2AK4 gene* mutations in PCH pathogenesis. However, it is likely that *EIF2AK4* mutations do not account for all cases of PCH [2].

In our case, CGHa revealed a chromosome 16q23.3-q24.1 deletion that disrupts the distant *FOXF1* transcriptional enhancer, which maps about 257 kb upstream to the *FOXF1* gene. In this enhancer are located two lncRNAs: *LINC01081* and *LINC01082* [7]. It has been demonstrated that *in vitro* abolition of *LINC01081* by siRNA, reduces *FOXF1* expression [14]. Thus, our data could suggest that the disruption of the *FOXF1* gene transcriptional enhancer induces cell proliferation and migration. This is consistent with other findings indicating *FOXF1* as an oncosuppressor gene [15, 16]. The analysis of fusion progeny between mesenchymal stem cells and lung cancer cells has recently demonstrated that *FOXF1* significantly reduced the growth rate and expression levels of proteins regulating the cell cycle [17]. Accordingly, previous investigations have proposed PCH to be a lung endothelial neoplasia [18]. Based upon the relevance of *LINC01081* on *FOXF1* expression regulation, our data could suggest that the disruption of this long non-coding RNA can lead to architectural changes in pulmonary vessels, resulting in neonatal-onset PCH.

## Consent

This study has been performed in accordance with the Helsinki declaration. Written informed consent was obtained from the parents of the patient for publication of this case report. A copy of the written consent is available for review by the Editor of this journal.

## Additional file

Additional file 1: Table S1. Genes involved in our PCH patient deletion. (DOCX 18 kb)
