# Supplementary material for: A 16q deletion involving FOXF1 enhancer is associated to pulmonary capillary hemangiomatosis
Source: BMC Med Genet. 2015 Oct 13;16:94. doi: 10.1186/s12881-015-0241-7 (PMC4605103; doi:10.1186/s12881-015-0241-7)
Supplement: Additional file 1: Table S1. — Genes involved in our PCH patient deletion. (DOCX 18 kb) [file 12881_2015_241_MOESM1_ESM.docx]

***Additional file 1: Table S1. Genes involved in our PCH patient deletion.***

| Gene | Chromosome position (by Ensembl) | Tissue expression (by Human protein atlas) | Function (by UniProtKB) | Disease association (by OMIM) | Inheritance |
| --- | --- | --- | --- | --- | --- |
| CDH13 | [82660408-83830204](http://grch37.ensembl.org/Homo_sapiens/Location/View?db=core;g=ENSG00000140945;r=16:82660408-83830204) | Expressed in skeletral, smooth and heart muscle | Calcium-dependent cell adhesion protein | None | / |
| HSBP1 | 83841448-83853342 | Widely expressed | Negative regulator of the heat shock response. Negatively affects HSF1 DNA-binding activity. May have a role in the suppression of the activation of the stress response during the aging process. | None | / |
| MLYCD | [83932731-83949787](http://grch37.ensembl.org/Homo_sapiens/Location/View?db=core;g=ENSG00000103150;r=16:83932731-83949787) | Widely expressed | Catalyzes the breakdown of malonyl-CoA to acetyl-CoA and carbon dioxide. Malonyl-CoA is an intermediate in fatty acid biosynthesis, and also inhibits the transport of fatty acyl CoAs into mitochondria. Consequently, the encoded protein acts to increase the rate of fatty acid oxidation. | Malonyl-CoA decarboxylase deficiency | AR |
| OSGIN1 | 83981887-83999937 | Widely expressed | Regulates the differentiation and proliferation of normal cells through the regulation of cell death. | None | / |
| NECAB2 | 84002237-84036381 | Selective expression in neuropil and subset of neurons in CNS. | No data available for Molecular function | None | / |
| SLC38A8 | 84043272-84076241 | Fetal and adult brain, spinal cord | No data available for Molecular function | Foveal hypoplasia 2, with or without optic nerve misrouting and/or anterior segment dysgenesis | AR |
| MBTPS1 | 84087368-84150511 | Widely expressed | Catalyzes the first step in the proteolytic activation of the sterol regulatory element-binding proteins | None | / |
| HSDL1 | 84155886-84178797 | Highly expressed in testis and ovary. | No data available for Molecular function | None | / |
| DNAAF1 | 84178865-84212373: | Ubiquitous | Cilium-specific protein required for the stability of the ciliary architecture. Plays a role in cytoplasmic preassembly of dynein arms. Involved in regulation of microtubule-based cilia and actin-based brush border microvilli. | Primary ciliary dyskinesia or Kartagener syndrome | AR |
| TAF1C | 84211458-84220669 | Ubiquitous | Component of the transcription factor SL1/TIF-IB complex, which is involved in the assembly of the PIC (preinitiation complex) during RNA polymerase I-dependent transcription. | None | / |
| ADAD2 | 84224744-84230774 | Widely expressed | No data available for Molecular function | None |  |
| KCNG4 | 84255823-84273356 | Expressed in Leydig cells of testis. | Potassium channel subunit that does not form functional channels by itself. Can form functional heterotetrameric channels with KCNB1; modulates the delayed rectifier voltage-gated potassium channel activation and deactivation rates of KCNB1 | None | / |
| WFDC1 | 84328252-84363450 | Widely expressed | Growth inhibitory activity | None | / |
| ATP2C2 | 84402133-84497793 | Widely expressed | Transports Ca(2+) and Mn(2+) into the Golgi lumen for protein sorting, processing, and glycosylation. It is also involved in Ca(2+) signaling, independent of its ATPase activity | None | / |
| TLDC1 | 84511681-84587639 | Widely expressed |  | None | / |
| COTL1 | 84599200-84651683 | Widely expressed with highest levels in placenta, lung, kidney and peripheral blood leukocytes and lower levels in brain, liver and pancreas. | This protein binds F-actin, and also interacts with 5-lipoxygenase, which is the first committed enzyme in leukotriene biosynthesis | None | / |
| KLHL36 | 84682131-84701292 | Ubiquitous | Probable substrate-specific adapter of an E3 ubiquitin-protein ligase complex which mediates the ubiquitination and subsequent proteasomal degradation of target proteins. | None | / |
| USP10 | 84733584-84813528 | Widely expressed | Hydrolase that can remove conjugated ubiquitin from target proteins such as p53/TP53, BECN1, SNX3 and CFTR. Acts as an essential regulator of p53/TP53 stability. | None | / |
| CRISPLD2 | 84853590-84954374 | Ubiquitous | Promotes matrix assembly. | None | / |
| ZDHHC7 | 85007787-85045141 | Widely expressed | Palmitoylacyltransferase that functions in palmitoylation of sex steroid receptors | None | / |
| KIAA0513 | 85061375-85127836 | Ubiquitous | No data available for Molecular function | None | / |
| FAM92B | 85131965-85146114 | Expressed in respiratory epithelia and uterus | No data available for Molecular function | None | / |
| GSE1 | 85645015-85709810 | Expressed in testis and smooth muscle. | No data available for Molecular function | None | / |
| GINS2 | 85709804-85723679 | Widely expressed | Plays an essential role in the initiation of DNA replication, and progression of DNA replication forks. | None | / |
| EMC8 | 85805364-85833214 | Expressed in liver, pancreas, heart, lung, kidney, brain, skeletal muscle, and placenta. | Component of the ER membrane protein complex (EMC) | None | / |
| COX4I1 | 85832239-85840650 | Ubiquitous | This protein is one of the nuclear-coded polypeptide chains of cytochrome c oxidase, the terminal oxidase in mitochondrial electron transpor | None | / |
| IRF8 | 85932409-85956215 | Predominantly expressed in lymphoid tissues | Specifically binds to the upstream regulatory region of type I IFN and IFN-inducible MHC class I genes and plays a negative regulatory role in cells of the immune system. | Immunodeficiency 32A, mycobacteriosis, | AD |
|  |  |  |  | Immunodeficiency 32B, monocyte and dendritic cell deficiency | AR |

AD= autosomal dominant; AR= autosomal recessive
